# Crimean–Congo haemorrhagic fever virus S-segment mRNA 5′ UTR functions as an internal ribosome entry site-like element in an N protein-dependent manner

**DOI:** 10.1099/jgv.0.002359

**Published:** 2026-10-27

**Authors:** Songyang Ren, Ethan Mir, Hoangvi Le, Saima Ali, Sheema Mir, Mohammad Mir

**Affiliations:** 1 College of Veterinary Medicine, Western University of Health Sciences, Pomona, California, USA; 2 Department of Microbiology, Immunology and Molecular Genetics, UCLA, Los Angeles, California, USA

**Keywords:** Crimean–Congo haemorrhagic fever virus, internal ribosome entry site (IRES), nucleocapsid protein, translation

## Abstract

The Crimean–Congo haemorrhagic fever virus nucleocapsid (N) protein contains structurally distinct stalk and head domains. The mRNA encoding N protein (NP) possesses a 55-nt 5′ UTR that folds into a stable hairpin structure. We previously demonstrated that the NP preferentially promotes translation of its own mRNA by simultaneously binding the 5′ UTR hairpin via its stalk domain and the translation initiation factor eIF4A via its head domain. This coordinated interaction facilitates selective ribosome recruitment to the 5′ UTR and enhances translation of the downstream ORF. In this study, we further show that the S-segment 5′ UTR functions as an internal ribosome entry site (IRES)-like element when inserted between two ORFs, promoting downstream translation in an NP-dependent manner. Expression of a dual-reporter transcript containing the S-segment 5′ UTR in Hazara virus-infected cells inhibited viral replication by competing for NP binding. In contrast, randomization of the 5′ UTR abolished NP binding, IRES-like activity and antiviral effects. Unlike the S segment, the L- and M-segment 5′ UTRs do not form the requisite hairpin structure, fail to bind NP and cannot support NP-mediated translation. These findings suggest that structurally similar elements within host mRNAs may also be selectively translated during infection.

## Introduction

Crimean–Congo haemorrhagic fever virus (CCHFV) is a tick-borne *Nairovirus* belonging to the order *Bunyavirales*. The viral disease is endemic across wide geographic regions, including Africa, the Balkans, the Middle East and parts of Asia, and is associated with case fatality rates of up to 40% [1–4]. Although CCHF is not currently endemic in the USA [5], it remains a potential public health concern due to international travel and the presence of competent tick vectors capable of transmitting the virus [6]. CCHFV is transmitted through tick bites, direct contact with blood or tissues from infected animals and person-to-person exposure via infected bodily fluids [7, 8]. There is currently no licensed vaccine, and treatment is largely supportive. The antiviral agent ribavirin has demonstrated some therapeutic benefit, and the development of mRNA-based vaccines has been proposed [9]. Recent increases in outbreaks, particularly in Mediterranean countries, are likely linked to the widespread distribution and expanding populations of the tick vectors, potentially influenced by climate change [10]. The CCHFV genome comprises three negative-sense RNA segments S, M and L, which encode the N protein (NP), the glycoprotein precursor and the RNA-dependent RNA polymerase (RdRp), respectively [11]. A defining feature of the bunyavirus genome is the presence of partially complementary sequences at the 5′ and 3′ termini. These sequences bp to form a panhandle structure that serves as the template for viral transcription and replication and plays a critical role in genome encapsidation and packaging. Recently, the X-ray crystallographic structure of CCHFV NP at 2.3 Å resolution revealed the presence of head and stalk domains in the NP structure [12–14]. The protein structure is more closely related to the nucleocapsid protein of Arenaviruses [15, 16] compared to the nucleocapsid protein of other Bunyaviruses [17–19]. We showed that the stalk domain harbours a dsRNA-binding site [20, 21] and the head domain harbours an ssRNA-binding site. Although NP is primarily seen as a crucial part of the viral capsid, recent research on the Lassa fever virus (Arenaviridae) and Sin Nombre hantavirus (Hantaviridae) has uncovered NP’s diverse roles in the viral replication cycle [15, 16, 22–24].

CCHFV initiates transcription through a cap-snatching mechanism in which the viral RdRp cleaves 10–14 nt from the 5′ termini of host cell mRNAs [25]. The resulting capped RNA fragments serve as primers for viral mRNA synthesis. These host-derived capped mRNA sequences remain fused with the 5′ UTR of the viral mRNA. We previously demonstrated that the CCHFV NP enhances translation of viral mRNA through interactions involving its 5′ UTR [25] and eukaryotic translation initiation factor eIF4A, a key component of the eIF4F complex that functions as the ribosomal recruitment platform [26].

In the present study, we further demonstrate that the S-segment mRNA 5′ UTR can function as an internal ribosome entry site (IRES)-like element when positioned between two ORFs, facilitating translation of the downstream ORF in an NP-dependent manner. This finding is significant because many host mRNAs contain long and structurally complex 5′ UTRs that may harbour similar hairpin elements. Such structures could potentially be recognized by the CCHFV NP in infected cells, leading to selective translational upregulation of specific host factors.

## Methods

### Cells and other reagents

BHK-21 cells were from Dr De La Torre’s lab (Scripps Research Institute, La Jolla, CA). Hazara virus was from the World Reference Center for Emerging Viruses and Arboviruses (WRCEVA) at UTMB. Monoclonal antibodies for actin (Cat# 3700S) and His-tag (Cat#12698S) were from Cell Signaling Technologies. Polyclonal anti-Hazara virus NP antibody was developed by ProSci. All other reagents including the TurboFect transfection reagent were from Fisher Scientific.

### Plasmids

The plasmid encoding the wild-type CCHFV NP from strain 10200 was originally provided by Dr Stuart T. Nichol (CDC, Atlanta, GA), as previously described [27]. The ORFs encoding the wild-type NP, head domain and stalk domain were PCR amplified and cloned in pTriEx1.1 backbone, as previously reported [20]. The dual-reporter construct (pcDNA-GFP-EMCV-IRES-mCherry) was generated by synthesizing a DNA fragment encoding the encephalomyocarditis virus (EMCV) IRES, positioned between the GFP and mCherry ORFs. The synthesized fragment was flanked by HindIII and NotI restriction sites (GenScript). UAG stop codons were introduced downstream of both the GFP and mCherry ORFs. The synthetic DNA fragment was digested with HindIII and NotI and ligated into the corresponding restriction sites of the pcDNA3.1(+) backbone. The same strategy was used for the construction of pcDNA-GFP-S-UTR-mCherry, pcDNA-GFP-M-UTR-mCherry and pcDNA-GFP-L-UTR-mCherry plasmids by incorporating the 5′ UTR sequences of CCHFV (Strain 10200) S-, L- and M-segment-derived mRNAs. The randomized S-segment 5′ UTR sequence was incorporated in pcDNA-GFP-S-Random UTR-mCherry plasmid.

### Immunoprecipitation

BHK-21 cells were transfected with the required dual-reporter plasmids. At 24 h post-transfection, cells were infected with Hazara virus at an m.o.i. of 0.1. Cells were harvested and lysed at 36 h post-infection. Immunoprecipitation was performed using an anti-NP antibody (ProSci), as previously described [28]. Briefly, cells were lysed in NP-40 lysis buffer (50 mM Tris-HCl, pH 7.5; 150 mM NaCl; 0.5% NP-40; 10% glycerol; 1 mM EDTA) supplemented with protease and phosphatase inhibitor cocktails (Roche). Clarified lysates were obtained by centrifugation, and 10% of the total lysate was reserved as input. The remaining lysate was incubated with 1 µg of the indicated antibody for 4 h at 4 °C with gentle rotation. Immune complexes were captured by incubation with 40 µl of protein G agarose beads (50% slurry) for 1 h at 4 °C with continuous rotation. The beads were washed four times with lysis buffer, resuspended in 1× SDS sample loading buffer and boiled at 95 °C for 5 min. Following brief centrifugation, the supernatants were resolved by SDS-PAGE and analysed by Western blotting using the appropriate antibodies.

### Real-time PCR

Hazara virus load in infected cells was determined by quantification of viral S-segment vRNA using real-time PCR, as previously described [20, 25, 28–30]. Briefly, total RNA was extracted from virus-infected and control cell lysates using the RNeasy Kit (Qiagen) and reverse transcribed with M-MLV Reverse Transcriptase (Invitrogen) according to the manufacturers’ instructions. Quantitative real-time PCR (qRT-PCR) was performed using the ABI 7500 Real-Time PCR System (Applied Biosystems) with SYBR Green PCR Master Mix (Roche), as previously reported [20, 25, 28–30]. *β*-actin mRNA was quantified as an internal control for normalization. Relative RNA levels were calculated using the comparative Ct (ΔΔCt) method as described previously [20, 25, 28–30]. The primer sequences used for amplification of *β*-actin mRNA and Hazara virus S-segment RNA have been reported previously [20, 25, 28–30]. Similarly, the dual-reporter mRNA levels in cell lysates were determined using a primer set targeted to the GFP ORF. To determine whether the dual-reporter mRNA co-purified with the NP in the immunoprecipitated material, cell lysates were subjected to immunoprecipitation as described above. Purified total RNA (1 µg) from BHK-21 cells was added to 50 µl of the immunoprecipitated material. Total RNA was then re-purified using the RNeasy Kit (Qiagen). Reporter mRNA levels were quantified by real-time PCR using a relative quantification method, with primers specific for GFP and *β*-actin as an internal control, as described above.

### Fluorescence microscopy

BHK-21 cells were co-transfected with plasmids expressing the NP, the stalk or head domain, along with the dual-reporter construct of interest. At 24–36 h post-transfection, cells were washed three times with 1× PBS and fixed with methanol. GFP and mCherry signals were then visualized using an Echo Revolve microscope (ECHO). GFP and mCherry fluorescence intensities were measured at multiple fields from the indicated slides using Fiji software. The fluorescence intensity values were averaged to determine the mean fluorescence intensity and sd for each sample. Statistical significance between groups was assessed by calculating *P* values using Student’s t-test.

### Statistical analysis

Each experiment was independently repeated at least three times. Data points were averaged to calculate the mean, with sd shown as error bars. Statistical significance between groups was determined by calculating *P* values using Student’s t-test. *P*<0.05 was considered significant, with significance indicated as *P*<0.05 (*), *P*<0.005 (**) and *P*<0.0005 (***).

### Chemiluminescence plaque assay

Hazara virus titres in the culture media were determined by plaque assay, previously used for Prospect Hill virus [28]. Briefly, the assay was performed according to the previously reported protocol, with the following modifications: SeaPlaque Agarose (Lonza, Cat. No. 50100) was used to prepare the overlay medium, and a rabbit polyclonal anti-Hazara virus NP antibody was used at a 1 : 50 dilution.

## Results and discussion

The rapid production of viral proteins and replication of the viral genome within host cells are critical for the efficient spread of newly formed virions to neighbouring cells. Accordingly, the efficiency of viral protein synthesis directly influences viral load in infected hosts and may contribute to disease severity. To optimize replication, viruses have evolved diverse strategies to enhance translation of their own mRNAs while minimizing competition with host transcripts for the cellular translation machinery [21]. For example, we previously demonstrated that the NP of hantaviruses, another member of the order Bunyavirales, specifically binds both the 5′ cap structure of mRNA and ribosomal protein S19, a component of the 40S ribosomal subunit [15, 22]. This interaction enables the hantavirus NP to recruit the 40S ribosomal subunit directly to the mRNA 5′ cap, independently of the canonical eIF4F cap-binding complex [15, 22]. The eIF4F complex, composed of eIF4A, eIF4G and eIF4E, normally mediates recruitment of the 43S preinitiation complex to the mRNA 5′ cap during host cap-dependent translation [23] (Fig. 1d). We further showed that this hantavirus-specific mechanism preferentially enhances translation of mRNAs containing the viral 5′ UTR [24].

**Fig. 1.**
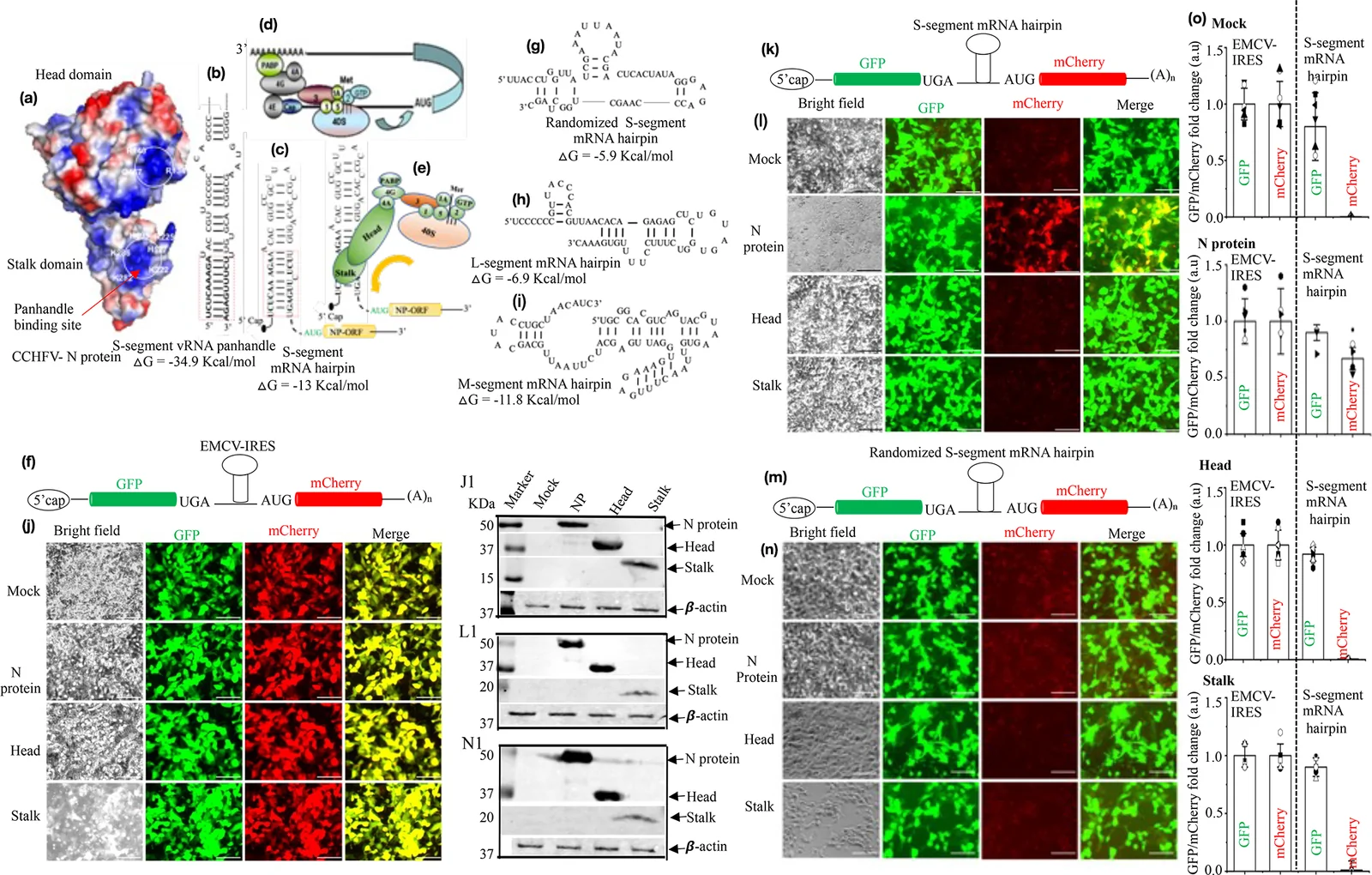
CCHFV S-segment mRNA 5′ UTR functions as an IRES: (**a**) modelled three-dimensional structure of the CCHFV NP highlighting the stalk and head domains. Electrostatic surface potential was visualized using PyMOL, with positively charged regions shown in blue and negatively charged regions in red. Discrete positively charged patches, formed by the indicated aa residues, represent putative RNA-binding pockets within both the head and stalk domains. (**b**) S-segment viral RNA (vRNA) panhandle structure of CCHFV. (**c**) Panhandle-like hairpin formed by the 5′ UTR of the CCHFV S-segment-derived mRNA. The conserved stem region (red box) is required for binding to the stalk domain of the NP. (**d**) Schematic of canonical eukaryotic translation initiation (several initiation factors were omitted for clarity). (**e**) Proposed model of CCHFV NP-mediated translation initiation. (**f**) Diagram of a dual-reporter mRNA containing the EMCV IRES positioned between GFP and mCherry ORFs. (g–i) Predicted secondary structures of (**g**) randomized CCHFV S-segment mRNA 5′ UTR, (**h**) L-segment-derived 5′ UTR and (**i**) M-segment-derived 5′ UTR. (**j**) Fluorescence microscopy of BHK-21 cells co-transfected with the dual-reporter construct expressing the mRNA shown in panel (f) and either an empty vector (mock) or plasmids expressing full-length CCHFV NP, the stalk domain or the head domain. (**k**) Schematic of a dual-reporter mRNA containing the CCHFV S-segment mRNA 5′ UTR inserted between GFP and mCherry ORFs. (**l**) Fluorescence microscopy of BHK-21 cells co-transfected with the construct expressing the mRNA shown in panel (k) and either an empty vector (mock) or plasmids expressing CCHFV NP, stalk domain or head domain. (**m**) Schematic of a dual-reporter mRNA containing a randomized CCHFV S-segment mRNA 5′ UTR between GFP and mCherry ORFs. (**n**) Fluorescence microscopy of BHK-21 cells co-transfected with the construct expressing the mRNA shown in panel (m) and either an empty vector (mock) or plasmids expressing CCHFV NP, stalk domain or head domain. (**j1, l1, n1**) Immunoblot analysis of lysates from cells shown in panels (j), (l) and (n), probed for expression of NP, head domain, stalk domain and *β*-actin using specific antibodies. *Note*: Scale bar in fluorescence images: 10 µm. (**o**) GFP and mCherry fluorescence intensities from each slide in panels (j) and (l) were measured at multiple fields using Fiji software. The intensity values were averaged and normalized to the corresponding mean values in panel (j). The resulting normalized values, along with their sd (error bars), are presented in panel (o). The significance of the mCherry signal difference (second and fourth bar from the left) revealed a *P* value of <0.00005 for mock, stalk and head domains. The *P* value for NP was <0.005. The *P* values were calculated using Student’s t-test.

The NP of CCHFV possesses a distinctive structural architecture comprising a stalk domain and a head domain (Fig. 1a). We previously demonstrated that the stalk domain specifically binds the panhandle structure formed by base pairing between the 5′ and 3′ termini of the viral genomic RNA (Fig. 1b). Terminal nine nt of the panhandle, boxed in Fig. 1(b), are required for high-affinity binding to the stalk domain. This stalk domain–panhandle interaction is unique to Nairoviruses and plays a critical role in genome encapsidation and packaging. Notably, the 5′ UTR of S-segment-derived mRNA also adopts a panhandle-like hairpin structure (Fig. 1c). The boxed nine nt required for high- affinity binding to stalk domain are conserved in both the vRNA panhandle and the hairpin structure of the 5′ UTR (see boxed region in Fig. 1b, c). Using multiple complementary experimental approaches, we previously showed that the NP simultaneously engages the 5′ UTR hairpin structure via its stalk domain and the translation initiation factor eIF4A via its head domain (Fig. 1e). The simultaneous binding facilitates ribose engagement on the viral mRNA 5′ UTR. In contrast to canonical host cap-dependent translation (Fig. 1d), this NP-mediated mechanism is dedicated to viral mRNA translation. We further demonstrated that CCHFV S-segment mRNA is heavily associated with ribosomes, with the NP likely remaining bound to the 5′ UTR to continuously promote ribosome recruitment, enhance polysome formation and drive efficient NP synthesis in infected cells [26].

To determine whether the 5′ UTR hairpin of the CCHFV S-segment mRNA can function as an IRES, we generated a dual-reporter construct in which wild-type S-segment 5′ UTR of the CCHFV strain 10200 (~50 nt) was inserted between the GFP and mCherry ORFs in the pcDNA3.1(+) backbone, as described in Methods (Fig. 1k). A second construct was generated in which the S-segment 5′ UTR sequence was randomized prior to insertion between the GFP and mCherry ORFs (Fig. 1m). As a positive control, a third construct was generated in which the EMCV IRES was inserted between GFP and mCherry (Fig. 1f). BHK-21 cells were co-transfected with the dual-reporter plasmid harbouring the EMCV IRES together with plasmids expressing either the full-length CCHFV NP or its stalk or head domains. Reporter expression was analysed 36 h post-transfection. As shown in Fig. 1(j), both GFP and mCherry, translated via cap-dependent initiation and the EMCV IRES-mediated mechanism, respectively, exhibited strong reporter signals. The wild-type CCHFV NP and its stalk and head domains were also robustly expressed from the respective plasmids (Fig. 1j). Importantly, co-expression of the NP or its domains had no detectable effect on GFP or mCherry expression (Fig. 1j), indicating that these viral proteins do not interfere with the host translation machinery.

The experiment was subsequently repeated under identical conditions, except that the dual-reporter construct harbouring either the wild-type (Fig. 1l) or randomized (Fig. 1n) 5′ UTR of the CCHFV S-segment mRNA was used for co-transfection. Reporter expression was assessed by fluorescence microscopy (Fig. 1l, n), while expression of the NP and its individual domains was confirmed by Western blot analysis (Fig. 1l1, n). As shown in Fig. 1(l), GFP expression was readily detected; however, mCherry expression was observed only when full-length NP was co-expressed. Co-expression of the stalk or head domains had no effect on GFP or mCherry expression, similar to mock-transfected cells (Fig. 1l). Interestingly, when the S-segment 5′ UTR was randomized, mCherry expression was abolished, and GFP expression remained unaltered, despite robust expression of the full-length NP (Fig. 1n, n1). The GFP and mCherry fluorescence signals in Fig. 1(l) were quantified and normalized to the corresponding signals obtained with the control construct shown in Fig. 1(j). The resulting normalized values were plotted in Fig. 1(o). This analysis revealed that the NP-mediated translational enhancement of mCherry with the assistance of wild-type S-segment 5′ UTR was ~70% of the enhancement observed with the EMCV IRES. These results demonstrate that the wild-type S-segment 5′ UTR specifically promotes translation of the downstream ORF in the presence of the NP. Quantification of reporter mRNA levels showed that differences in reporter expression were not due to changes in mRNA levels (data not shown).

The findings in Fig. 1 are consistent with our previous observations demonstrating that the S-segment 5′ UTR of CCHFV forms a stable hairpin structure (Fig. 1c), in contrast to the randomized UTR (Fig. 1g). The conserved base-paired stem within this hairpin is required for high-affinity binding by the NP stalk domain, and nt randomization abolishes the stem structure and impacts the RNA–protein interaction. Although the stalk domain alone retains RNA-binding capacity, it lacks the head domain necessary for interaction with eIF4A and subsequent ribosome recruitment. Conversely, the head domain cannot bind the RNA hairpin independently, indicating that both domains are required for functional translation activation. In contrast to the S-segment UTR, the 5′ UTRs of the L and M segments are longer but fail to form a comparable stable secondary structure and lack the conserved stem required for NP stalk binding. Consistent with this, we previously showed that bacterially expressed NP does not bind the L- or M-segment UTRs with high affinity [26]. We therefore hypothesized that these UTRs would not function in NP-mediated translation. To test this, L- and M-segment 5′ UTRs were inserted between GFP and mCherry in the dual-reporter constructs (Fig. 2a, c) and evaluated in co-transfection assays. Neither construct supported mCherry expression in the presence of full-length NP or its individual domains (Fig. 2b, d), indicating that only the S-segment 5′ UTR confers NP-dependent translational activity.

**Fig. 2.**
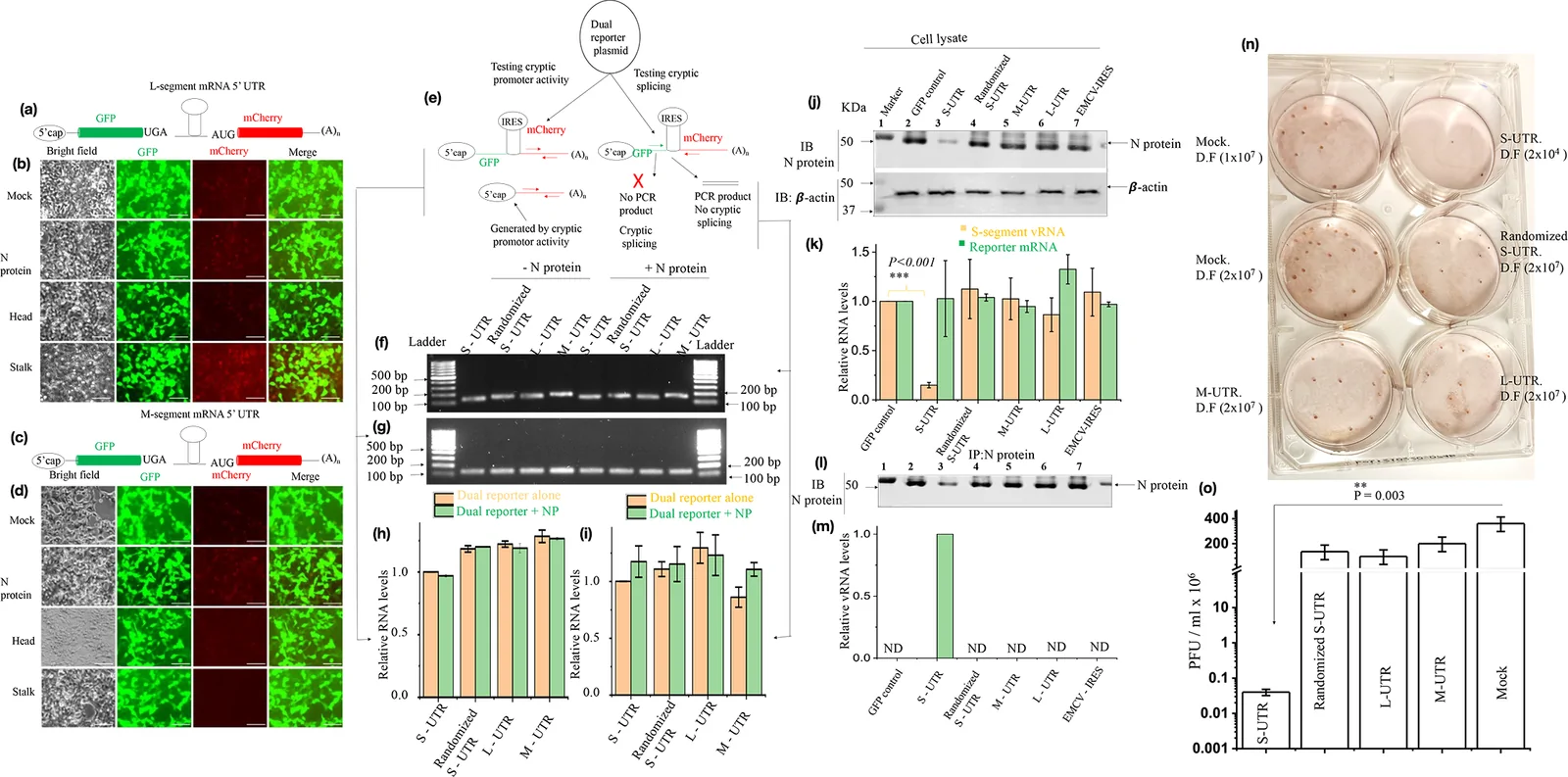
Impact of dual-reporter mRNA expression on Hazara virus replication in cell culture: (**a**) schematic of a dual-reporter mRNA containing a randomized CCHFV L-segment mRNA 5′ UTR between GFP and mCherry ORFs. (**b**) Fluorescence microscopy of BHK-21 cells co-transfected with the construct expressing the mRNA shown in panel (a) and either an empty vector (mock) or plasmids expressing CCHFV NP, stalk domain or head domain. (**c**) Schematic of a dual-reporter mRNA containing a randomized CCHFV M-segment mRNA 5′ UTR between GFP and mCherry ORFs. (**d**) Fluorescence microscopy of BHK-21 cells co-transfected with the construct expressing the mRNA shown in panel (c) and either an empty vector (mock) or plasmids expressing CCHFV NP, stalk domain or head domain. (**e**) A schematic showing the strategy used to test the cryptic promoter activity and cryptic splicing of dual-reporter mRNA. (f, i) A primer set complementary to GFP and mCherry as shown in panel (e) was used in the real-time PCR analysis of the dual-reporter mRNA, (**i**) and the PCR products were separated on the gel (**f**). (g, h) A primer set complementary to mCherry as shown in panel (e) was used in the real-time PCR analysis of the mRNA, (**h**) and the PCR products were separated on the gel (**g**). (**j**) BHK21 cells were transfected with either GFP expression construct (GFP control) or dual-reporter constructs harbouring either S-UTR or randomized S-segment UTR or M-UTR or l-UTR or EMCV-IRES, followed by Hazara virus infection, as mentioned in the text. Cell lysates were examined for the expression of Hazara virus NP or *β*-actin using appropriate antibodies. (**k**) Total RNA was purified from a portion of the cell lysates from panel (j), followed by quantification of hazara virus S-segment vRNA (yellow bars) or reporter mRNA (green bars) by realtime PCR, using appropriate primer sets. (**l**) Half of the lysates from panel (j) was immunoprecipitated using an anti-Hazara virus NP antibody, followed by Western blot analysis of the immunoprecipitated material using the same antibody. (**m**) Total RNA was purified from the immunoprecipitated material shown in panel (g), followed by quantification of dual-reporter mRNA using primer pairs targeted to GFP. (**n**) BHK-21 cells were transfected with dual-reporter constructs, followed by Hazara virus infection as mentioned in panel (j). The media harvested from infected cells 48 h post-infection were examined by plaque assay, as mentioned in Methods. (**o**) The foci in panel (n) were used to calculate viral titres p.f.u. ml^−1^ and plotted versus the corresponding treatment. *Note*: D.F in panel (n) stands for dilution factor and mock represents the untransfected control.

We next investigated whether the mCherry signal observed in NP-expressing cells (Fig. 1l) resulted from cryptic splicing of the dual-reporter mRNA at or downstream of the S-segment 5′ UTR, or from cryptic promoter activity within the S-segment UTR that could generate an mCherry-encoding transcript. BHK-21 cells were cotransfected with plasmids expressing wild-type NP and a dual-reporter construct containing either the S-, L- or M-segment 5′ UTR, or a randomized S-segment 5′ UTR (Fig. 2e). To assess the possibility of cryptic splicing, mRNA was reverse transcribed, and the resulting cDNA was subjected to PCR using primers complementary to the GFP and mCherry ORFs that flank the UTR sequence (Fig. 2e). The expected PCR product was detected (Fig. 2f), indicating that the dual-reporter mRNA remained intact and ruling out cryptic splicing in cells.

We next examined whether NP expression altered the abundance of the dual-reporter mRNA. Real-time PCR analysis showed that the dual-reporter mRNA was expressed at comparable levels from the transfected plasmids in the absence and presence of NP (Fig. 2i). These results are consistent with the comparable GFP expression observed from the dual-reporter mRNA under both conditions (Fig. 1l). Finally, we tested whether the mCherry signal in NP-expressing cells could result from cryptic promoter activity within the S-segment UTR. Such activity would be expected to generate both the full-length dual-reporter mRNA and a separate transcript encoding mCherry alone (Fig. 2e). However, real-time PCR using primers specific for the mCherry ORF revealed comparable levels of mCherry-containing transcripts in cells in the absence and presence of NP (Fig. 2g, h). These results rule out the generation of an additional mCherry-encoding transcript through cryptic promoter activity and further support the conclusion that the observed mCherry signal is not due to aberrant transcription or processing of the dual-reporter mRNA. However, although less likely, it remains unclear whether other alternatives, such as ribosomal readthrough or reinitiation downstream of the S-segment 5′ UTR, could facilitate mCherry expression in NP-expressing cells.

To further validate the high-affinity interaction between the NP stalk domain and the S-segment 5′ UTR and to establish its functional relevance in translation control, we examined whether the CCHFV S-segment 5′ UTR hairpin expressed from the dual-reporter construct could compete with Hazara virus S-segment mRNA during infection. Notably, the 5′ UTR of the Hazara virus S-segment mRNA also adopts a hairpin structure containing the conserved base-paired stem required for NP binding, in a Nairovirus-specific manner.

BHK-21 cells were transfected with dual-reporter constructs harbouring the wild-type CCHFV S-segment 5′ UTR, a randomized S-segment UTR, L- or M-segment-derived UTRs or an EMCV IRES and were infected 24 h later with Hazara virus, a BSL-2 model for CCHFV. Both viral genomic RNA and reporter mRNA levels were quantified by real-time PCR. NP expression was evaluated by Western blotting. As shown in Fig. 2(k), reporter mRNAs were expressed at comparable levels in all samples. However, both NP expression (Fig. 2j) and viral S-segment RNA levels (Fig. 2k) were markedly reduced in cells expressing the dual reporter containing the CCHFV S-segment 5′ UTR, indicating inhibition of viral replication. To determine whether this inhibition resulted from sequestration of Hazara virus NP by the hairpin structure of CCHFV S-segment mRNA 5′ UTR, cell lysates were subjected to immunoprecipitation using an anti-NP antibody (Fig. 2l), followed by RNA purification and qRT-PCR analysis. Only the reporter mRNA containing the CCHFV S-segment 5′ UTR co-purified with NP, despite its reduced steady-state abundance in lysates (Fig. 2m). These findings demonstrate that the CCHFV S-segment 5′ UTR hairpin competitively binds Hazara virus NP, thereby limiting its availability for Hazara virus replication. In contrast, nt randomization of the S-segment UTR, as well as L- and M-segment-derived UTRs lacking the conserved stem, abolished NP binding and competitive inhibition. To further confirm the inhibition of Hazara virus replication in cells expressing the dual-reporter mRNA containing the S-segment 5′ UTR, cells were transfected with the indicated dual-reporter constructs and subsequently infected with Hazara virus, as described above. Viral titres in the culture media were then determined by plaque assay. As shown in Fig. 2(n, o), expression of the dual-reporter mRNA containing the S-segment 5′ UTR resulted in a significant reduction in viral titres. These results are consistent with the findings shown in Fig. 2(j, k) and further confirm that the presence of the S-segment 5′ UTR in the dual-reporter mRNA inhibits Hazara virus replication.

## Concluding remarks

Taken together, our findings indicate that Nairoviruses have evolved a conserved regulatory strategy centred on a stable, base-paired stem within the vRNA panhandle and S-segment mRNA 5′ UTR. As previously reported, this structure is specifically recognized by the RNA-binding site in the stalk domain of the NP. The resulting RNA–protein interaction plays multiple roles in the viral life cycle, most notably by promoting selective translation of S-segment mRNA through ribosome engagement at its 5′ UTR. When positioned between two ORFs, the S-segment 5′ UTR functions as an IRES-like element in an NP-dependent manner. Although speculative, this mechanism may extend beyond viral RNAs. Host mRNAs harbouring structurally similar hairpins within their complex 5′ UTRs could potentially be translationally enhanced in infected cells. Consistent with this concept, we recently showed that hantavirus NP promotes translation of the proviral host factor valosin-containing protein via its 5′ UTR, and this upregulation is required for efficient hantavirus assembly, egress and spread [28]. Collectively, these observations define a conserved NP-mediated translational control mechanism and suggest that the CCHFV S-segment 5′ UTR could be harnessed as a synthetic regulatory element to enhance protein expression in the presence of NP.

## Abbreviations

CCHFV: Crimean–Congo haemorrhagic fever virus
EMCV: encephalomyocarditis virus
IRES: internal ribosome entry site
NP: N protein
qRT-PCR: quantitative real-time PCR
RdRp: RNA-dependent RNA polymerase
